# Interchangeable utilization of metals: New perspectives on the impacts of metal ions employed in ancient and extant biomolecules

**DOI:** 10.1016/j.jbc.2021.101374

**Published:** 2021-10-31

**Authors:** Daniel G.J. Smethurst, Natalia Shcherbik

**Affiliations:** Department for Cell Biology and Neuroscience, School of Osteopathic Medicine, Rowan University, Stratford, New Jersey, USA

**Keywords:** metals, iron, manganese, magnesium, ribosome, superoxide dismutase, reactive oxygen species, redox regulation, metalloprotein, interchangeability, cam-SOD, cambialistic SOD, Co, cobalt, COVID-19, coronavirus disease 2019, Cu, copper, Fe, iron, Fe–S, iron–sulfur, Fe-SOD, Fe-containing SOD, H_2_O_2_, hydrogen peroxide, ISCU, Iron–Sulfur Cluster, MCO, metal-catalyzed oxidation, MCT, metal chelation therapy, Mg, magnesium, Mn, manganese, Mn-SOD, manganese-containing SOD, Ni, nickel, O_2_•^−^, superoxide, •OH, hydroxyl radical, RdRp, RNA-dependent RNA polymerase, RNR, ribonucleotide reductase, ROS, reactive oxygen species, Rpe, ribulose-5-phosphate 3-epimerase, SARS-CoV2, severe acute respiratory syndrome coronavirus 2, SOD, superoxide dismutase, ZF, zinc finger, Zn, zinc

## Abstract

Metal ions provide considerable functionality across biological systems, and their utilization within biomolecules has adapted through changes in the chemical environment to maintain the activity they facilitate. While ancient earth's atmosphere was rich in iron and manganese and low in oxygen, periods of atmospheric oxygenation significantly altered the availability of certain metal ions, resulting in ion replacement within biomolecules. This adaptation mechanism has given rise to the phenomenon of metal cofactor interchangeability, whereby contemporary proteins and nucleic acids interact with multiple metal ions interchangeably, with different coordinated metals influencing biological activity, stability, and toxic potential. The ability of extant organisms to adapt to fluctuating metal availability remains relevant in a number of crucial biomolecules, including the superoxide dismutases of the antioxidant defense systems and ribonucleotide reductases. These well-studied and ancient enzymes illustrate the potential for metal interchangeability and adaptive utilization. More recently, the ribosome has also been demonstrated to exhibit interchangeable interactions with metal ions with impacts on function, stability, and stress adaptation. Using these and other examples, here we review the biological significance of interchangeable metal ions from a new angle that combines both biochemical and evolutionary viewpoints. The geochemical pressures and chemical properties that underlie biological metal utilization are discussed in the context of their impact on modern disease states and treatments.

The unique chemical properties of numerous metal ions facilitate extensive interactions with biomolecules, with impacts across all areas of cellular activity, including fundamental processes, such as respiration, metabolism, nitrogen fixation, photosynthesis, DNA replication, transcription, and protein synthesis (1, 2, 3, 4, 5, 6, 7, 8). At least ten metal elements are considered essential for most forms of life (9), including six of the d-block elements of the periodic table: manganese (Mn), iron (Fe), cobalt (Co), nickel (Ni), copper (Cu), and zinc (Zn) ((10) and Fig. 1*A*). These metals are characterized by the ability to form ions with partially filled d-subshells (shown in Fig. 1*A*, *blue*). This electron configuration facilitates multiple oxidation states, defining many of their chemical properties. These metals are called transition metals, with the most biologically relevant examples appearing in the first row of the d-block in the periodic table (Fig. 1*A*, *dark cyan squares*). Although biologically important, Zn is excluded from the transition metals by some definitions because of possession of a complete d-subshell (Fig. 1*A*).

**Figure 1:**
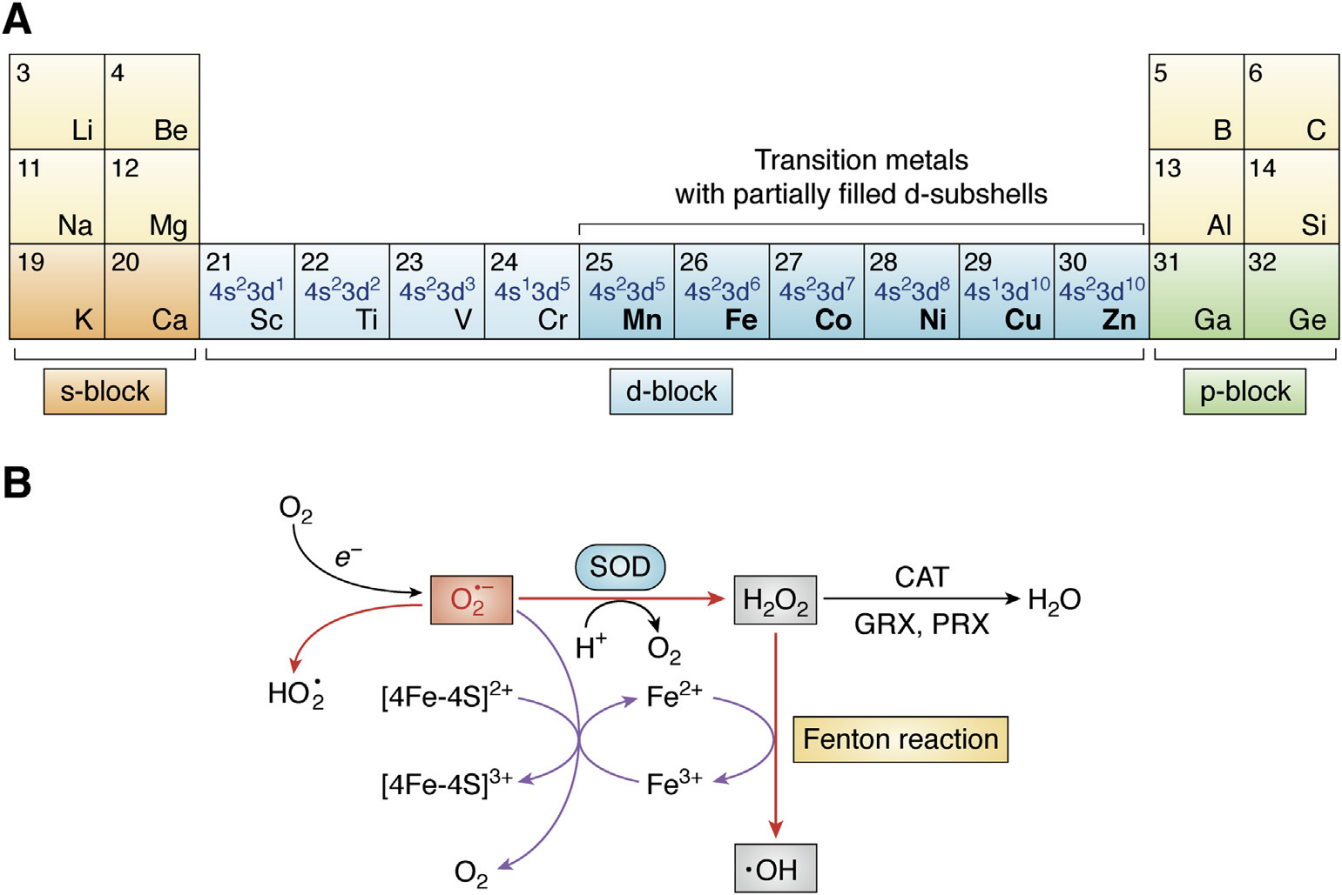
**Transition metals and oxidative stress.***A*, transition metals. A section of the periodic table showing s-block (*orange*), d-block (*cyan*), and p-block (*green*) chemical elements. Transition metals of the first row, characterized by partially filled d-subshells, are shown in *dark cyan squares* in *bolded black lettering*. Zn (which possesses a complete d-subshell) is also shown in this group. The electronic structures of the d-block elements are shown in *blue lettering*. *B*, reactions of the superoxide anion; iron and redox cycling. Negatively charged free radical superoxide (O_2_^•−^, shown in *red lettering*) is the product of one-electron (*e*^*−*^) reduction of dioxygen (O_2_). Upon protonation, O_2_^•−^ can form the hydroperoxyl radical (HO_2_^•^). Superoxide dismutase (SOD, *cyan oval*) catalyzes the dismutation (disproportionation) of O_2_^•−^, thereby generating O_2_ and hydrogen peroxide (H_2_O_2_). H_2_O_2_ is converted to H_2_O by various antioxidant enzymes, such as catalases (CAT), glutathione peroxidases (GPX), and peroxiredoxins (PRX). Redox-active Fe^2+^ ions are oxidized by H_2_O_2_, generating highly reactive hydroxyl radicals (OH^•^) and Fe^3+^ through the Fenton reaction. Fe^3+^ can be reduced to Fe^2+^ by O_2_^•−^, resulting in redox cycling (*purple arrows*). By itself, O_2_^•−^ can reduce Fe^3+^ to Fe^2+^ within iron–sulfur cluster proteins, resulting in enzyme inactivation and accumulation of Fe^2+^, which further powers Fenton chemistry. Modified from Ref. (271).

The human body contains amounts of magnesium (Mg), Fe, and Zn in the gram range, whereas milligram amounts of Mn, Cu, Co, and molybdenum are present (9, 11). These metals perform both catalytic and structure-stabilizing roles and are predominantly available as divalent cations (possessing two fewer electrons than the neutral state). In fact, approximately 40% of enzymes with known structures depend upon at least one metal cofactor for catalytic activity (12). Analysis of metal-binding domains in the proteome suggests that metal-mediated folds are proportional to proteome size across the kingdoms of life, whereas the specific metals predicted to be utilized reveal distinct changes through evolutionary history (13). The utilization of metal ions for life on earth may predate protein-oriented extant biology, as nucleic acids in a metal-rich prebiotic environment are hypothesized to generate the earliest enzymatic mechanisms. Much as in proteins, metal ions are employed as catalytic cofactors in RNA species (14) and coordinated by both the anionic sugar phosphate backbone and the nucleotide bases (4, 14). Metal ions similarly interact with the DNA backbone and bases (15). The high charge density of the metal ions allows large RNAs to form complexes and closely packed folds and tertiary interactions, facilitating elaborate and dynamic structures, such as the ribosome—the essential protein-synthesizing machine that operates in every living cell. Transition metal ions bind throughout rRNA both loosely and at specific sites, with Mg being the major metal ion contributing to present structures of large and small ribosomal subunits (4, 14, 16).

Despite an effort, drawing definitive conclusions about the physiological utilization of metal ions by biomolecules within cells is a technically challenging task, hindered by several experimental issues. These include the dissociation of ions during biomolecule purification, physical properties that limit detection in structural models, and broader impacts of overexpressing metal-binding species on metal availability (17). Further complications in the elucidation of metal ions usage for biomolecules functionality arise from the phenomenon of metal ion interchangeability, wherein one of several different ions are able to occupy a specific biomolecular binding site. Metal ions, in many cases, self-assemble into complexes with biomolecules (18) and, as such, can be greatly influenced by intracellular metal availability dictated by a particular physiological or environmental condition. Therefore, the flexibility in metal ion preference may be more prevalent than currently understood. Thus, the view of a single native metal for a given binding site may, at least in some cases, be an unfavorably strict categorization. In a healthy cell, cytosolic and organellar metal cation levels are tightly regulated as a means of protection against the undesired activity of certain metal elements, while metal imbalances manifest in numerous human diseased states. This identified link between the ability of certain biomolecules to interact with a non-native metal ion(s), which may abolish their correct functionality leading to mitigation of disease, supports the need for further knowledge of the molecular mechanisms governing metal ion interchangeability. Such investigation will advance our understanding of disease etiology and progression, with a potential for new therapeutical intervention strategies.

Particular progress in revealing transition metal interchangeability in biomolecular structure and function has been made recently because of new technological developments. An emerging example of particular interest is the utilization of metals by the ribosome. While the high Mg content of contemporary ribosomes supports a preference of the metal-binding sites for Mg^2+^, a recently published study that replicated a prebiotic environment of anoxic earth rich in Fe and Mn and low in oxygen revealed that Mg^2+^ on ribosomes can be replaced by Fe^2+^ or Mn^2+^ without affecting protein-synthesizing activity (19, 20). Besides an important impact on new biochemical features of a ribosome and medicine-related translational science, this technically advanced approach provided the scientific community with an elaborate biological model crucial for investigating the origin of life and evolution of biological molecules. In fact, the recapitulated conditions mimicking the anoxic earth environment supported a hypothesis that a ribosome represents an extraordinarily well-conserved RNA–protein structure that existed in a complex with Fe ions when earth's atmosphere was depleted of oxygen (3, 20). These studies were corroborated with biochemical assays conducted with ribosomes from *Saccharomyces cerevisiae*, wherein it was demonstrated that eukaryotic ribosomes maintained an ability to interact with Fe^2+^ at the selected sites under normal physiological conditions. This suggests a possibility that this biological atavism might play an essential role in the regulation of protein synthesis, the faultless processivity of which contributes to protein homeostasis and protection against neurodegenerative diseases (21). These recent developments in understanding ribosome biology in the context of transition metal interchangeability open up many questions requiring further investigation.

Flexibility in metal ion interactions with biomolecules is not only limited to ribosomes but also has been documented to be prevalent in metalloproteins. This is commonly observed in *in vitro* assays, wherein enzyme activity is assessed in the presence of different metals to identify which confers the greatest catalytic activity. In addition, certain metal ions, including Mg, are not directly detectable in spectroscopic studies and difficult to identify in crystallographic studies (22, 23) and can be readily substituted with alternative ions for the purposes of structural assessment (24, 25), further indicating the relative ease with which certain ions can replace one another.

Large-scale environmental changes accompanied with the accumulation of molecular oxygen in ancient earth's atmosphere, the groundbreaking event that occurred in the course of evolution dated billions of years ago, drove adaptation of biomolecules by selecting organisms that had the capability to defend against highly reactive chemical products derived from the incomplete reduction of oxygen (known as reactive oxygen species [ROS]) and utilize alternative metals to catalyze crucial biochemical reactions. One of the prominent examples of how these pressures are mirrored in extant biology is the host–pathogen interface. The innate immune system orchestrates challenging chemical assaults upon invading pathogens that in many cases display flexibility in metal utilization in their responses. For example, the connection between metal biochemistry and oxidative stress is central to the phagocytic immune response, which employs both oxidant assault and nutrient metal limitation in the defense against pathogens (26). Many pathogens are sensitive to metal levels on either side of a relatively narrow window, and host systems exploit this (27). During infection of a host organism, bacterial pathogens will commonly experience challenging conditions, including both overload and limitation of trace metal elements, as well as severe oxidative stress (28, 29, 30).

Transition metals are also known to play an important role in several viruses' survival and pathogenesis. Relevant to the present time, much of the research has been conducted investigating the role of Fe and other transition metals in the severe acute respiratory syndrome coronavirus 2 (SARS-CoV2)–related pathologies. Although many questions remain unanswered, it is clear that metals play an important role during SARS-CoV2 infection and propagation, whereas multiple manifestations of coronavirus disease 2019 (COVID-19), such as immune dysfunction, inflammation, hypercoagulation, hyperferritinemia, have been linked to Fe overload (31).

The possible ambiguity in assignments of metal ion associations with biomolecules, along with emerging examples of physiologically relevant metal interchangeability, indicates an existing gap in knowledge with an impact on diverse biological topics briefly mentioned previously. To gain an up-to-date picture of the transition metal interchangeability phenomenon, here, we discuss the current progress that has been made.

As such, we outline the deeply embedded nature of divalent metal cations in biological macromolecules and place this in the context of the risk of oxidative damage, which is present in an oxygen-rich environment. The geochemical changes that occurred on earth since the establishment of life are then discussed as they relate to metal availability and utilization. As some of the best-studied examples that have had recent developments in understanding metal interchangeability, we describe the role of metal ions in the superoxide dismutases (SODs), the R2 subunit of the ribonucleotide reductases (RNRs), and the ribosome. We choose these biomolecule examples as they remain in the frontline of scientific research, providing new information on how metals can replace each other to meet various physiological cues. Finally, we describe how flexible metal utilization impacts both bacterial and viral infections and immunity. While we focus on Fe and Mn as the most prominent examples of physiological metal cofactors, other biologically important metals are also discussed.

## Metal ions provide powerful biochemical functionality to biomolecules

As stated previously, ∼40% of biomolecules utilize metals as cofactors, suggesting that metal ions are essential for cellular physiology, with the first-row transition metals being of particular importance (Fig. 1*A*). For their interactions with biomolecules, metal ions can be considered in terms of several properties, including charge density, radii, and reactivity. Redox activity is also of particular relevance to biology because it prescribes some catalytic capabilities. Redox inactive metal cations, of which Mg and Zn are the most common in biomolecules, tend to be utilized in structures to stabilize negative charges, as well as functioning as Lewis acids to activate substrates by accepting lone pair electrons with no net change in oxidation state (12, 32, 33). Many of the transition metals are redox active, such as Fe, Mn, Cu, and Ni, as electrons of their incomplete d-subshells (Fig. 1*A*) can be lost, allowing for several oxidation states. While such cations can act as Lewis acids, they are commonly used in the catalysis of redox reactions, in which electron transfer results in a change of oxidation state.

Both the intracellular availability and the stability of formed complexes are important factors in metal cofactor binding. The predicted complex stability of divalent metal cations is described by the Irving–Williams series (Mg^2+^ < Mn^2+^ < Fe^2+^ < Ni^2+^ < Co^2+^ < Cu^2+^ > Zn^2+^) (34), which illustrates that stability of complexes increases with atomic number across the divalent metal cations until reaching Zn, which does not possess unpaired d-shell electrons and thus forms less stable interactions than Cu^2+^ (35). However, many biomolecules associate with less competitive cations, such as Mg^2+^, Fe^2+^, and Mn^2+^ (18), as well as monovalent cations of potassium or sodium, which form even less stable interactions.

Despite tight regulation of cellular concentrations of Mg^2+^, Fe^2+^, and Mn^2+^ accomplished by a coordinated effort of metal transporters and buffering chaperones in regulating free ion levels (36), the high affinity of these metals toward biomolecules ensures their activity even at low concentrations. For example, Fe is central to the heme and Fe–sulfur (Fe–S) cluster complexes, which are crucial cofactors in electron transport chain reactions, oxygen transport, translation termination, and antioxidant pathways. The background and current understanding of Fe–S cluster assembly and function have been detailed in several informative recent reviews (37, 38). The synthesis of these ancient cofactors can be catalyzed by UV light from the reduced Fe–S species, which were prevalent in the prebiotic earth, supporting an early role for Fe–S clusters in the evolution of life (39).

The propensity for biomolecules to be flexible in their metal binding partner may reflect two features of the interactions. First, while biomolecular structures may evolve to prefer a metal cofactor by excluding similar ions, which are suboptimal, nonfunctional, or deleterious, there are overlapping characteristics of cations, which in many cases impede absolute specificity. Second, experimental evidence of selective advantages gained by retaining or acquiring the ability to exchange cofactors suggests that tolerance of alternative metal ions may be beneficial in certain circumstances. In other examples, divergent or convergent evolution has produced multiple distinct biomolecules within organisms, allowing consistent activity in environments of varying metal availability and limitations.

## Change of earth's atmosphere as a driving force of the evolution of biomolecules

### Great oxidation events, molecular oxygen, and ROS

Early in the history of earth, volcanic processes were the major contributors to the composition of the atmosphere and oceans. This ancient earth's atmosphere was anoxic and reducing, with oceans rich in soluble divalent transition metal ions (40, 41, 42). While the time line of changes in earth's atmosphere remains under discussion (43, 44), it is thought that 2 to 3 billion years ago, the accumulation of molecular oxygen in the atmosphere occurred in what is known as the great oxidation event. A further oxidation event is likely to have transpired less than 1 billion years ago (neoproterozoic oxidation event), which had a more significant impact on oxygen levels in the ocean (45). These shifts in atmospheric composition occurred subsequent to biogenesis (44, 45), involving complex fluctuations of oxygen stores with biological processes likely being the primary source of the molecular oxygen (43, 46, 47). It has been proposed that prevalent methanogenic archaea, which depended on Ni for metabolic catalysis, declined following a decrease in volcanic sources of Ni (48). This led to a decrease in methane production and allowed proliferation of species requiring less Ni, such as photosynthetic marine cyanobacteria (40, 49).

Abundant oxygen led to the expansion of organisms utilizing oxidative metabolism, with oxygen acting as an electron acceptor, as it contains two unpaired electrons with parallel spins and is metabolized by a univalent (single electron) mechanism, generating several ROS as intermediates. Among others (50), ROS include the superoxide (O_2_^•−^), hydroxyl (•OH) radicals, and hydrogen peroxide (H_2_O_2_) (51). H_2_O_2_, while itself not a radical, is prone to univalent reduction by Fe and Cu ions, making it a significant contributor to oxidative damage to various biomolecules, along with the O_2_^•−^ and •OH radicals. O_2_^•−^ radicals are generated from the electron transport chain reactions and are converted to the less reactive H_2_O_2_ by SODs (43). Other antioxidant enzymes, such as catalase, glutathione peroxidase, and/or peroxiredoxins then convert H_2_O_2_ to water (52). Alternatively, the •OH radical can be generated from H_2_O_2_ by high-energy radiation, or by metal ion catalysis reactions, such as the Fenton reaction that is discussed later (Fig. 1*B*).

### Oxidation of biomolecules and role of metal ions

ROS, especially highly reactive •OH radical, can oxidize most biological targets, resulting in short lifetimes and very limited diffusion distances (53). ROS broadly damage proteins and amino acids *via* a range of modifications to amino acid side chains that lead to protein inactivation and degradation, the induction of polypeptide cleavages, and promotion of cross-linking and aggregation (54). In many cases, damage to the proteins can occur following exposure to xenobiotic metals, such as heavy metals (55). Another metal-induced harmful protein modification mechanism is related to site-specific metal-catalyzed oxidation (MCO) of amino acids at metal-binding sites. Specifically, MCO systems were found to target a wide variety of essential cellular enzymes and structural proteins, including glutamine synthetase, chymotrypsin, myosin, α-synuclein, catalases, and SODs. It was found that the activity of most of the MCO systems is dependent on ions of Fe or Cu, both of which are also involved in Fenton chemistry, whereby Fe^2+^, and in some instances, Cu^2+^, react with H_2_O_2_ to produce a •OH radical and a hydroxide ion ((56) and Fig. 1*B*). Fenton-generated •OH radical damages amino acids within proteins (57, 58).

The oxidative damage of proteins is implicated in a plethora of human pathologies, including neurodegenerative diseases, such as Alzheimer's (59) and Parkinson's disease (60), amyotrophic lateral sclerosis (61); muscular dystrophy (62), pulmonary emphysema (63), atherosclerosis (64), and age-related clinical pathologies, such as age-related macular degeneration (65), and cataractogenesis (66). For example, inactivation of SODs *via* oxidation (67, 68) or mutations (61, 69) results in enzymatic incompetency, degradation, or aberrant cellular localization. This leads to increased levels of ROS, causing protein damage and aggregation (which manifest in the progression of amyotrophic lateral sclerosis, Alzheimer's disease, and Parkinson's disease (70)) and DNA lesions (which can promote tumorigenesis (71)). Oxidation and cleavage of protein chaperones, such as Hsp90, has also been demonstrated to occur by an Fe-mediated mechanism, compounding the direct damage of ROS to proteins by disrupting the folding and stability of the targets of this chaperone (72, 73), leading to neurodegeneration and prionopathies (74). The scope of protein oxidation and the various mechanisms are reviewed in detail in Ref. (53).

Nucleic acids are also highly susceptible to damage by ROS, and oxidant damage to both RNA and DNA is implicated in many diseases, including neurodegenerative conditions and cancer (75, 76). The Fe- and/or Cu-driven Fenton reaction (Fig. 1*B*) has been identified as a source of oxidants leading to nucleic acid damage (77, 78, 79), and this is discussed in the following section. In addition, Fe and Mg can cleave RNA by a nonoxidative mechanism (termed in-line cleavage), as was recently demonstrated (3).

Oxidation of free RNA species can cause strand cleavages and oxidative base modifications. While oxidized mRNAs are recognized by ribosomes, the lesions are associated with decreased translational efficiency and an increase in truncated or misfolded protein products (80, 81). Besides damages to the transcripts, the translational machinery, which is constructed of proteins and RNAs, is also susceptible to oxidant-induced impairments. Ribosomes are large ribonucleotide–protein complexes that are at the center of the protein synthesis machinery. Ribosomes are composed of the small subunit (30S for prokaryotes and 40S for eukaryotes) and large subunit (50S for prokaryotes and 60S for eukaryotes). The subunits are assembled as intricately folded rRNAs for which Mg^2+^ ions play a critical role by coordinating rRNA folding and interaction with ribosomal proteins. Eukaryotes also have distinct ribosomes in the plastids and mitochondria, where they are likely to be particularly exposed to oxidative species derived from electron transport chain reactions (82). RNA oxidation leads to guanine modification (8-oxo-7,8-dihydroguanine), which is associated with wide range of pathologies, such as neurodegeneration, neuropsychiatric disorders, and atherosclerosis (82). Thus, oxidative stress negatively impacts translational processes (83), while maintenance of translational function can promote adaptation and survival responses (84, 85).

### Fenton chemistry and role of Fe and Cu

Fe can act as an electron donor and acceptor owing to its reduced Fe^2+^ and oxidized Fe^3+^ states, which are important for its biological utility. The employment of Fe in biomolecules became established in biology in an aqueous earth environment, which was Fe rich and contained little oxygen.

The mononuclear or dinuclear ions of Fe are employed in the catalytically active centers of many enzymes, including the SODs and RNRs, both of which are susceptible to metal interchange and are the focus of this review. Much of the toxicity associated with Fe is a result of the Fenton reaction (Fig. 1*B*). In the reducing environment of the cytosol, O_2_^•−^ can oxidize and destabilize Fe–S cluster complexes with crucial activity in the citric acid cycle, thereby blocking aerobic metabolism and generating free Fe, which can further participate in Fenton chemistry (Fig. 1*B*). The term “redox cycling” is used to refer to this propagation of ROS by a combination of the Fenton reaction and the activity of the O_2_^•−^ anion in the regeneration of free Fe^2+^ (51, 86).

Similar to Fe, ions of Cu that exist in oxidized (Cu^2+^) and reduced (Cu^+^) states play roles in electron transport and many redox enzyme–driven mechanisms (87). Importantly, Cu^+^ also participates in Fenton-like reactions, and there is a partial overlap of biological activities and interactions between homeostatic mechanisms between Cu and Fe. Both are highly redox active, facilitating roles in many enzymatic reactions (88). Because of the oxidative damage associated with loss of homeostasis of these metals, regulation of free Fe and Cu in cells is important for protection from metal-dependent oxidative damage. Cu is especially toxic because of subsequent reactions involving the Cu^2+^ ion, including binding to thiol groups, generating large amounts of •OH radicals, and promoting further Fe^2+^-mediated Fenton reactions (89). For these reasons, free Cu is extremely limited within cells, being almost entirely bound by highly conserved Cu-binding proteins (90). Toxicity of Cu is mitigated in cells by metallothioneins that sequester Cu as well as Zn and non-nutrient heavy metals cadmium and mercury (91). Cu was likely not employed in primitive biomolecules because of limited availability but became required later, for example, in the catalytic center of certain SOD enzymes (Table 1), as an alternative to Fe or Mn (87).

**Table 1:** Metal ions interchangeability within SODs

Kingdom/order	Species	SOD	Native Me cofactor	Active site's Me replacement	Dismutation enzymatic activity	Cambialism	Reference
Bacteria/Bacillales	*Staphylococcus aureus*	SodA	Mn^2+^	—	Active	—	(141)
		SodM^a^	Mn^2+^/Fe^2+^^a^	Mn^2+^ or Fe^2+^^a^	Active^a^	Yes^a^	(141)^a^
		smSod^a^	Mn^2+^^a^	Mn^2+^ or Fe^2+^^a^	Active^a^	Yes^a^	(257, 258)^a^
		stSod^a^	Mn^2+^^a^	Mn^2+^ or Fe^2+^^a^	Active^a^	Yes^a^	(258, 259)^a^
Bacteria/Enterobacterales	*Escherichia coli*	SodA	Mn^2+^	—	Active	—	(125, 260)
				Fe^2+^	Gain of function: peroxidase^c^	No	(141)
		Mutant SodA^G165T^^a^	—a	Fe^2+^^a^	Active^a^	Yes^a^	(139)^a^
		SodA	Mn^2+^	Mn^2+^, Fe^2+^ hybrid^b^	Partially active^b^	No^b^	(261)^b^
		SodB	Fe^2+^	—	Active	—	(125)
				Mn^2+^	Inactive	No	(125)
		Mutant SodB^T165G^^a^	—a	Mn^2+^^a^	Active^a^	Yes^a^	(137)^a^
		SodC	Cu^2+^Zn^2+^	—	Active	—	(262)
Bacteria/Streptomycetales	*Streptomyces coelicolor*	SodN	Ni^2+^	Ni^2+^	Active	—	(196, 197)
				Fe^2+^	Inactive	No	(197)
				Zn^2+^	Inactive	No	(197)
				Fe^2+^, Zn^2+^ hybrid^b^	Active^b^	No^b^	(196)^b^
Bacteria/Bacteroidales	*Porphyromonas gingivalis*	pgSod^a^	Fe^2+^^a^	Mn^2+^ or Fe^2+^^a^	Active^a^	Yes^a^	(258)^a^
Bacteria/Actinobacteria	*Propionibacterium shermanii*	psSod^a^	Fe^2+^^a^	Mn^2+^ or Fe^2+^^a^	Active^a^	Yes^a^	(258)^a^
Archaea/Sulfolobales	*Acidianus ambivalens*	FeSod	Fe^2+^	—	Active	—	(263)
				Co^2+^	Inactive	No	(263)
				Ni^2+^	Inactive	No	(263)
				Mn^2+^	Inactive	No	(263)
Fungi/Saccharomycetales	*Saccharomyces cerevisiae*	Sod1	Cu^2+^Zn^2+^	—	Active	—	(264)
		Sod2	Mn^2+^	—	Active	—	(265)
				Fe^2+^	Inactive	No	(136, 266)
Animalia/Mammalia	Mammals	SOD1	Cu^2+^Zn^2+^	—	Active	—	(190)
				Cu^2+^Cu^2+^	Inactive	No	(189)
		SOD2	Mn^2+^	—	Active	—	(120, 267)
				Fe^2+^	Gain of function: peroxidase^c^	No	(133)
		SOD3	Cu^2+^Zn^2+^	—	Active	—	(118, 267, 268, 269)
Plantae/laurales	*Cinnamomum camphora*	FeSOD^a^	Fe^2+^^a^	Mn^2+^ or Fe^2+^^a^	Active^a^	Yes^a^	(270)^a^

a Highlight SODs with cambialistic properties.

b Highlight hybrid SODs, whereby two different metal ions (indicated in the table) occupy two distinct enzyme dimer's subunits.

c Highlight SODs with peroxidase activity gained upon metal ion replacement.

## Chemical properties of Fe, Mn, and Mg dictate their interchangeability within biomolecules

Conserved and ancient biomolecules are associated with Mn and Fe as a result of the availability of these cations in the early earth environment, as well as the catalytic capabilities supplied by their redox activities, which provide essential functions. The utilization of either Fe or Mn within closely related SODs (Table 1) from the most ancient group of these enzymes reflects the chemical similarities of these two divalent ions for use in redox mechanisms. These transition metal elements are adjacent in the periodic table and have similar radii, ligand affinities, and coordination preferences, presenting a challenge for biomolecules to choose between (36, 92).

**Figure 2:**
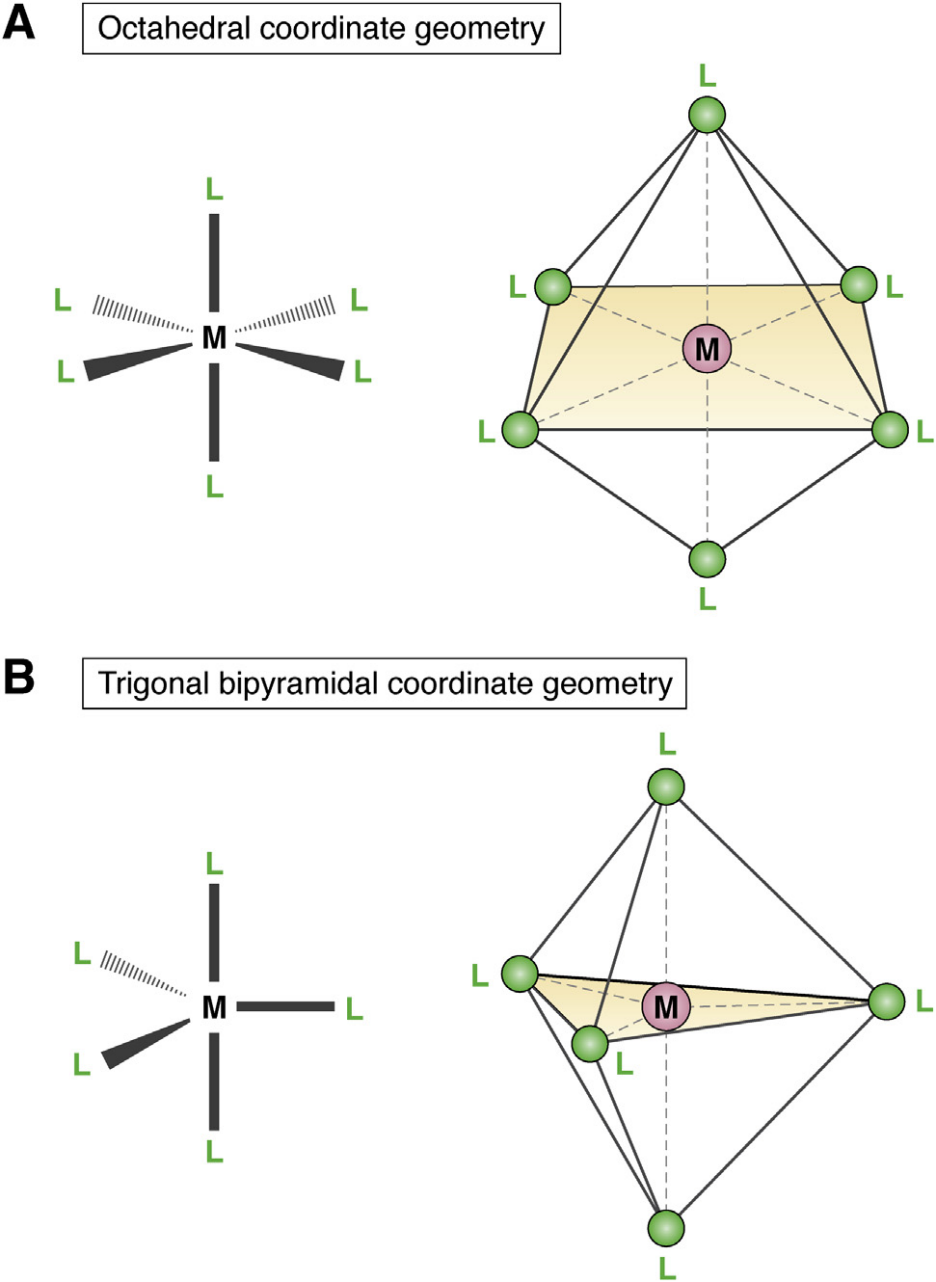
**The six- and five-coordinate geometries.** Diagrams (2D on the *left*; 3D on the *right*) of the six-coordinate octahedral geometry (*A*) and the atypical five-coordinate trigonal bipyramidal geometry (*B*) of various metal ions within biomolecules discussed in the article. M represents the ion of iron or manganese; L represents a ligand. Modified from Ref. (128).

Unlike Fe, Mn does not participate in Fenton chemistry because of possessing a higher reduction potential (93), and as such, does not present the same toxicity risks in an oxidative environment. Mn also functions in enzymes as a Lewis acid in mechanisms that are more comparable to those catalyzed by Mg or Zn (94). Coordinated Mg ions can often be replaced with Mn (2, 95, 96), as they have similar binding site requirements (97). While Mg is an alkaline metal possessing no d-electrons, the Mg^2+^ ion shares a relatively similar ionic radius to Mn^2+^ and Fe^2+^. When forming complexes, all three cations prefer to coordinate six ligands (24) in an octahedral liganding geometry (Fig. 2*A*), and, in several examples, can occupy the same binding sites (19, 22, 25, 98). Mg forms very few covalent interactions with its ligands, making it able to be rapidly exchanged. The d-electrons of Mn^2+^ contribute to electrophilic interaction with ligands, allowing it to tolerate greater distortions of the bonding geometry than Mg^2+^, thereby lending itself better to catalytic mechanisms (99). While the Irving–Williams series predicts greater stability of Mn or Fe complexes, the substitution of Mg with either transition metal may be limited by the vastly greater intracellular availability of Mg. In support of this, it has been reported that disruption of Mg^2+^-dependent processes occurs with increased Mn^2+^ import under conditions of low environmental Mg (96).

Mg is fundamental in stabilizing protein, lipid, and nucleic acid structures (100) and is involved in many catalytic mechanisms (25). It is the most common metal found in enzymes according to systematic analyses of reported protein structures, appearing in 16% of all enzymes (12, 100). For comparison, Mn is identified as a cofactor in approximately 6% of all enzymes with known structure, whereas Fe is a cofactor in 8% (12, 101). As Mg is known to form less stable interactions than other metal ions and is readily replaced, it is possible that structural reports underestimate the physiological occupation of binding sites by this element (101). Zn is also utilized extensively in cells and appears in more enzyme structures than Mn or Fe (101). Divalent ions of Zn and Mn have very similar radii (0.74 and 0.75 Å, respectively) but otherwise have dissimilar binding profiles and biochemical behavior and do not typically replace one another (24).

## Geochemical shifts drove extant metal biology: SOD as a prominent example

With the proliferation of oxygen and the potential for oxidative damage to cellular components by ROS, organisms that had evolved antioxidant defenses would have had a major advantage (43). Phylogenetic analysis suggests that ROS scavenging enzymes, such as SODs, peroxiredoxins, and catalases, had already emerged prior to the great oxidation event in response to the presence of low-level or localized oxygen (102). Antioxidant systems, thus, became increasingly valuable as oxygenation increased (43), whereas broad fluctuations in metal availability occurred concomitantly. The transition metals Mn, Fe, Co, and Ni were in solutions at relatively high levels in the early ocean environments because of the high sulfur content and low levels of oxygen, whereas precipitation of Cu and Zn would have rendered them unavailable for biochemistry (103). The increase in environmental oxygen led to the precipitation of Mn, Fe, Co, and Ni, and a major increase in the amount of available Zn, effectively reversing the availability of these metals (13, 42, 103, 104). These shifts in metal solubility were reflected by changes in their biological utilization. As such, comparison of structural motifs in the proteomes of organisms across the three domains of life supports the hypothesis that bioavailability of metals presented an evolutionary pressure resulting in the differences in their utilization (105).

**Figure 3:**
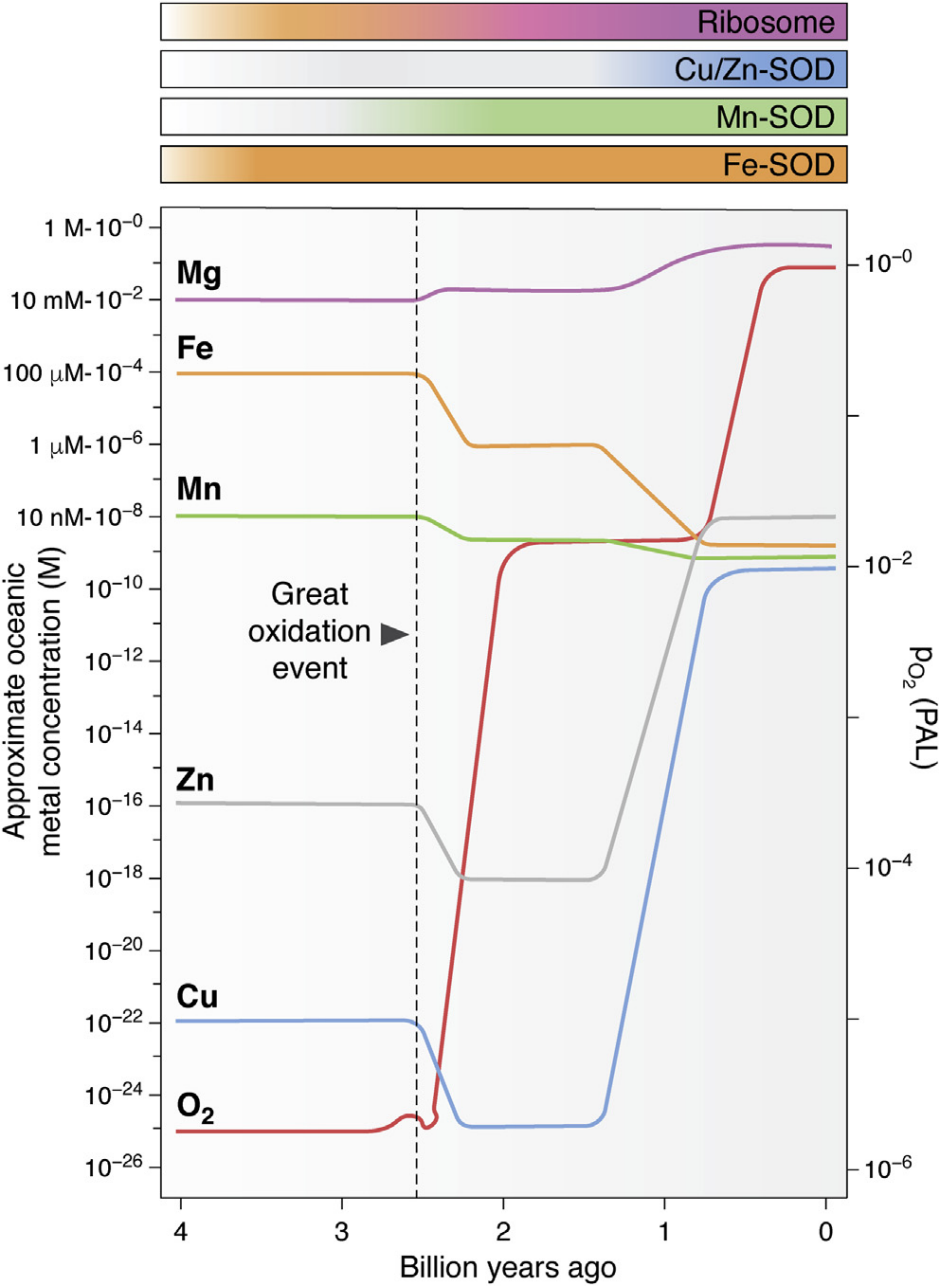
**Schematic representation of concentrations of oxygen and selected metal cations in earth oceans.** Estimates of the appearance of metal-utilizing biomolecules are shown above the graph in *gradient-colored horizontal bars*, indicating the early appearance of the ribosome. The most ancient superoxide dismutases (SODs), likely Fe-SODs, gave rise to Mn-SODs as oxygenation increased, with Cu/Zn-SODs appearing subsequently as copper and zinc became available. Oxygen levels (*red line*, *right axis*) are given as p_O2_ (partial pressure of oxygen) relative to PAL (present atmospheric level). The *dotted line* indicates the time of the great oxidation event (GOE). Modified from Refs. (103, 173, 272).

One prominent example of geochemical shift–driven evolution of biomolecules is the appearance of alternatives to the primitive Fe-containing SOD (Fe-SOD), which utilize other metal cofactors (Fig. 3 and Table 1). SODs are the major enzymes responsible for the removal of O_2_^•−^ anions that are unavoidably generated during aerobic metabolic reactions (51). British biochemist and writer Dr Lane characterized discovery of SODs as “*the most important discovery of modern biology never to win a Nobel prize*.” In fact, being discovered in 1969, SODs from various organisms remain of high interest for over last 5 decades.

The SODs catalyze a dismutation (disproportionation) reaction to produce O_2_ and H_2_O_2_ from two O_2_^•−^ *via* a cyclic oxidation–reduction electron transfer (106):

(1) $${\text{M}}^{\text{ox}}-\text{SOD}+{{\text{O}}_{2}}^{\text{•}-}{+\text{H}}^{+}⇄{\text{M}}^{\text{red}}-\text{SOD}\left({\text{H}}^{+}\right){+\text{O}}_{2}$$

(2) $${\text{M}}^{\text{red}}-\text{SOD}\left({\text{H}}^{+}\right)+{{\text{O}}_{2}}^{\text{•}-}{+\text{H}}^{+}⇄{\text{M}}^{\text{ox}}{-\text{SOD}+\text{H}}_{2}{\text{O}}_{2}$$

In this two-step reaction, the oxidized form of the metal (M) center ions (M^ox^-SOD) are first converted to the reduced form (M^red^-SOD) with the formation of O_2_ (reaction 1), followed by oxidation of the reduced form of the metal ions into their oxidized form by O_2_^•−^ with the release of H_2_O_2_ (reaction 2). Thus, one O_2_^•−^ reduces the SOD, whereas another O_2_^•−^ oxidizes the SOD in a so-called “ping–pong” mechanism (107). Several metal ion cofactors (such as Fe, Mn, Cu, Zn, and Ni) can be employed in the SOD active sites, which perform this mechanism, as summarized in Table 1. In this catalytic mechanism, the metal ion is utilized by the SODs as a source of protons by employing structural aspects of the metal binding site to adjust the redox potential, which also acts to regulate the access of anions (108). The activity of SODs requires them to exert tight control over the reactivity of the bound metals. The reduction midpoint potential (Em, a measure of the propensity of a chemical species to gain electrons in a redox reaction) of the metal cofactors is manipulated to around 300 mV in all known examples, despite the variety of metals employed across the enzyme families. The calculated reduction midpoint potential in aqueous solution compared with normal hydrogen electrode (a standard for zero redox potential as defined by the potential of a platinum electrode in 1 M acid solution) for the M^3+^/M^2+^ transition of Fe, Mn, and Ni are considerably different (0.77, 1.5, and 2.4 V, respectively) (109), requiring structural adaptation to bring the value into effective catalytic range (108, 110). The SODs demonstrate both the power of redox-active inorganic cofactors and the need to control their redox activity for function.

SODs are found in the archaea, prokarya, and eukarya ((43) and Table 1). Originally derived independently as three distinct families, abundance of SODs has undergone shift during course of evolution because of changes in earth's environment (Fig. 3). The first Mn/Fe-SOD arose in the low oxygen/high Fe environment (Fig. 3, *orange and green curves*). Thus, SODs represent a powerful and informative model to investigate metal ions interchangeability within biomolecules.

The Mn/Fe-SOD family utilizes Fe or Mn at their catalytic center, reflecting the high levels of these metals in the preoxidation earth in which they originated. Fe-SODs are found in some primitive eukaryotes, plants, and bacteria, whereas Mn-containing SODs (Mn-SODs) are widespread. Within bacteria, aerobes tend to contain Mn-SODs or both Mn-SOD and Fe-SOD, whereas strict anaerobes may have one Fe-SOD, or none at all (111). Mn-SOD (SodA) from *Escherichia coli* is flexible in binding Mn^2+^ or Fe^2+^ depending on growth conditions. As such, Mn-SOD prefers cognate Mn ion when cultivated in the presence of oxygen; whereas under anaerobic conditions, bacterial Mn-SOD accommodates ions of Fe resulting in dismutation-inactive enzyme. Excess Fe has also been linked to the formation of partially active Fe-Mn-SOD (hybrid SOD), wherein a dimer's subunits contain Mn^2+^ and Fe^2+^ in their active site resulting in partial activity (summarized in Table 1). Thus, it was proposed that the selectivity of a metal ion cofactor in the bacterial Fe/Mn-SODs is defined by the bioavailability of Mn or Fe (112).

The most recently evolved family, the Cu/Zn-SODs, utilize Cu along with Zn. The Cu/Zn-SODs are found only in certain bacterial species (113, 114) but are ubiquitous among higher eukaryotes (115, 116, 117). Humans express three Cu/Zn-SOD isoforms; the cytosolic SOD1 (117), the extracellular SOD3 (118), and the mitochondrial Mn-SOD (SOD2) (119, 120). They are absent in archaeal species and are considered to have appeared long after the evolution of the Mn/Fe-SOD family (107), correlating with the increase in bioavailable Cu and Zn (43, 103). The Ni-SODs are found in algae and predominantly marine species of bacteria and likely appeared after Mn/Fe-SODs, presenting a selective advantage in marine environments, as levels of Fe and Mn have diminished and concentrations of Ni remained relatively consistent (103, 121).

These examples illustrate that the appearance and accumulation of molecular oxygen throughout earth's evolution accompanied by a shift in the availability of transition metals induced a switch from Fe-containing SODs to new variants of this enzyme that adapted alternative metal ions as cofactors, as illustrated in Figure 3 (44, 122). Thus, the utilization of metal ions throughout extant biological systems is intrinsically linked to the employment of molecular oxygen for fundamental processes in aerobic biology and the management of the associated toxicity of ROS. In addition, extant systems can be informative of the chemical environments in which they arose, even when these are drastically different from those in which they now function.

## Cation replacement examples: Focus on Fe, Mn, and Mg

The exchange of Fe and Mn in functional enzymes is illustrated by a few prominent examples. The mononuclear SODs demonstrate the flexible utilization of either Mn or Fe within an isoform, whereas the essential RNR enzyme family, which employs a dinuclear cation pair, includes a further interesting example of an ancient and conserved enzyme, which utilizes different metal ions, and the expression of distinct forms with differing specificities. Finally, recent studies have identified an ability of the ribosome to undergo interchange of bound metal ions (20, 123). These examples, discussed in detail later, suggest that the transition metal interchangeability occurs across different molecular structures and can be informative about the conditions under which they developed.

### Mn-SOD, Fe-SOD, and cambialistic Mn/Fe-SOD

**Figure 4:**
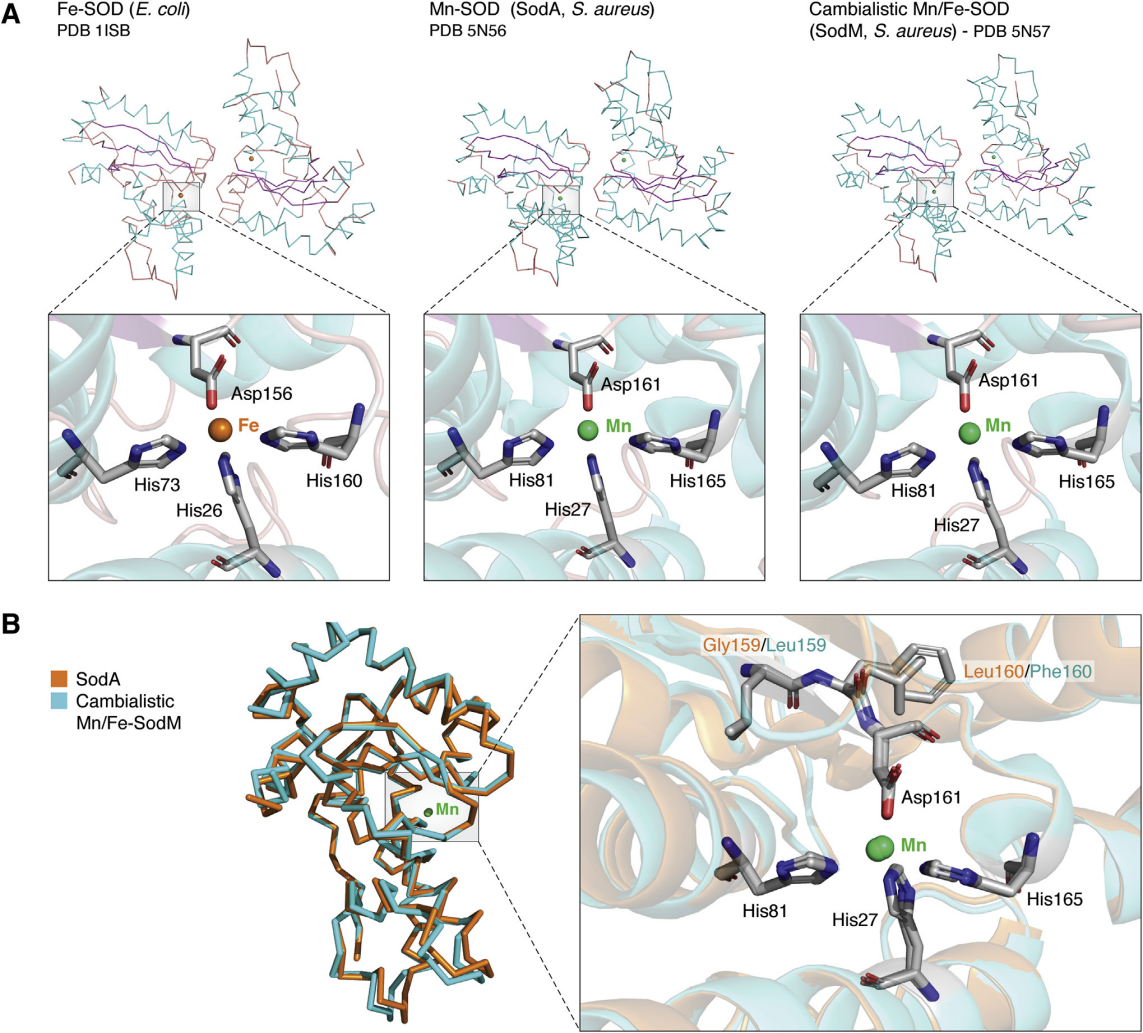
**Structural properties of Mn-specific, Fe-specific, and cambialistic Mn/Fe-SODs.***A*, comparison of the crystal structures (*top*) and the metal-bound active sites (*bottom*) of Fe-SOD from *Escherichia coli*, Mn-SOD (SodA) and cambialistic Mn/Fe-SOD (cam-SOD, SodM) from *Staphylococcus aureus*. Metal ions (shown as *colored spheres*; *orange* for iron ion and *green* for manganese ion) are coordinated by three histidine (His) residues and one aspartic acid (Asp) residue and a solvent molecule (not shown). The figure is generated using PyMol; Protein Data Bank IDs are indicated in the figure. *B*, superimposed ribbon representation of Mn-specific SodA (*orange*) and cambialistic Mn/Fe-SodM (*cyan*) monomer structures from *Staphylococcus aureus* is shown on the *left*. Superimposed structures of active centers of SodA and SodM are shown on the *right*. Four residues from the primary coordination sphere (His27, His81, Asp161, and His165, *black lettering*) coordinate a metal ion (Mn is shown as a *green sphere*). Two residues from the secondary coordination sphere (SodA: Gly159, Leu160, *orange lettering*; SodM: Leu159, Phe160, *cyan lettering*) provide cambialistic properties to SodM. SOD, superoxide dismutase.

The Fe-SODs and Mn-SODs are highly homologous in sequence and three-dimensional structure (124, 125), whereby the amino acid residues and the funnel that allows a substrate (O_2_^•−^) access to the metal ion are identical. Catalytically active sites of both SODs contain three histidines and one aspartic acid that bind the metal ion cofactor (Fig. 4*A*, *left and middle panels*). In addition, the Fe and Mn ions also bind to a solvent molecule (water or hydroxide) that is engaged in the formation of hydrogen bonds and, together with histidines and aspartic acid, participates in the coordination of either cation in an unusual distorted trigonal bipyramidal geometry around the metal center, as depicted in Figure 2*B* (126, 127, 128).

Despite this remarkable similarity, most Fe-SOD and Mn-SOD enzymes are only functional when bound to their cognate metal ion (Table 1), illustrating their high specificity to metal cofactor (129, 130, 131). Moreover, it has been revealed that the replacement of Mn with Fe in Mn-SODs from mammals (SOD2) and *E. coli* (SodA) results in the generation of an alternative isoform (Fe-SOD2s) with peroxidase prooxidant activity, thereby promoting oxidative stress, presumably *via* utilization of H_2_O_2_ ((132, 133) and Table 1). These findings demonstrate that, in some cases, incorporation of a noncognate metal ion does not disable an enzyme but switches its function, highlighting the biological significance of the metal selectivity process.

What is the mechanism behind the metal ions selectivity of SODs? Being structural components of SODs, Fe and Mn ions cycle between +2 and +3 oxidation states during O_2_^•−^ turnover; however, these oxidation states correspond to different d-electron configurations for Mn and Fe, resulting in distinct +3 to +2 reduction characteristics. It is believed that these differences are compensated by protein components, such as specific amino acids of a secondary coordination sphere of Fe-SOD or Mn-SOD. Of note, the first (inner) coordination sphere refers to the array of direct interactions of a ligand with a metal ion, whereas the secondary (outer) coordination sphere consists of ions that interact with the first coordination sphere, without direct binding to a metal ion. Although Mn-SODs and Fe-SODs share identical metal ion–containing catalytically active sites (Fig. 4*A*, *left and middle panels*), different amino acid residues lie adjacent to it and are responsible for metal ion specificity and activity that occur without major structural reorganization of the active center. For example, glutamine from the second coordination sphere of SODs was found to be involved in determining the redox potential of the active site, thus, impacting the metal cofactor selection process. As such, Gln69/Fe-SOD and Gln146/Mn-SOD from *E. coli* promote metal ion specificity (130, 131, 134, 135), and a similar observation was made of Sod2 (Mn-Sod) from *S. cerevisiae* (136). Furthermore, the crystal structures of native Mn-Sod2 and artificial Fe-Sod2 from *S. cerevisiae* at 2.05 and 1.79 Å resolution, respectively, demonstrated no significant alteration in the active site or overall structure upon binding the non-native metal Fe, and identified Asp163 and Lys80 as those responsible for the metal specificity of Mn-SOD (136). Another residue important for the SOD specificity is Thr165 present in Fe-SODs from *E. coli* (whereas a Gly residue occurs at this position in the majority of Mn-SODs (137, 138)), whereby swapping Thr165 and Gly165 in Fe-SOD (SodB) and Mn-SOD (SodA) changes the metal cofactor preference ((137, 139) and Table 1).

Despite the high metal ion specificity of Mn-SODs and Fe-SODs, some organisms have acquired an alternative form of an enzyme that can accommodate Mn or Fe ions in the catalytic center and retain enzymatic activity. These metal cofactor flexible SODs have been termed cambialistic or cam-SOD (140). Cam-SODs were found in microorganisms adapted to different growth conditions, including microaerophiles, aerobes, obligate anaerobes, and thermophiles. This interesting phenomenon raises questions of how and why some of these enzymes developed cambialistic properties.

In this regard, SodM (cam-SOD) from the Gram-positive opportunistic pathogen *Staphylococcus aureus* has become an informative experimental model, as it allowed direct comparison with the strictly Mn-dependent SodA (Mn-SOD) isoform (141, 142, 143). Thus, it has been shown that Mn- and Fe-bound cam-SOD exhibits comparable enzymatic activities (141). Moreover, the X-ray diffraction analysis performed with Mn-loaded or Fe-loaded SodA and SodM demonstrated only minor deviations in the catalytic center architecture and metal binding physicochemical properties, whereby metal ions (Mn or Fe) are coordinated by His27, His81, Asp161, and His165 ((144) and Figure 4*A*, *middle and right panels*). These structural similarities suggest that cambialism is not provided by the inner sphere coordination geometry but relies on differences in the secondary coordination sphere. Mutational analysis has identified two amino acids present in positions 159 and 160 (Fig. 4*B*) that vary between SodA (possesses Gly159 and Leu160) and SodM (Leu159 and Phe160) and make no direct contact but are in close proximity (<10 Å) to a metal cofactor (144). Significantly, swapping these amino acids between SodA and SodM did not affect active center structures but enabled cambialistic properties to SodA (144). It was proposed that amino acid side chains in positions 159 and 160 are responsible for changes in the reduction potential of the metal ions, likely underlying the mechanism of catalysis governed by Mn and Fe ions (144). This example indicates that subtle sequence alterations near the active site impose metal specificity on one isoform or allow flexibility in the other. One possible explanation for the impact of the amino acid residues equivalent to 159 and 160 of *S. aureus* is their role in assembling the appropriate hydrogen-bonding network that includes a metal-coordinated solvent, as described in Ref. (139). However, other studies demonstrated that solvent proton positions are similar in the structure of Mn-SOD and cam-SOD (145). These discrepancies call for further evaluation of the mechanisms by which the secondary sphere amino acids control redox tuning in cooperation with Mn and Fe ions.

The key Leu159 and Phe160 residues of cam-SODs are highly conserved within the lineages of the *S. aureus* tree, including *Staphylococcus argenteus* and *Staphylococcus schweitzeri* (146). Thus, it has been proposed that cam-SOD arose from a redundant gene encoding a second Mn-SOD *via* evolutionary-enforced mutagenesis (144, 147). Such an Fe-to-Mn switch likely occurred in response to diminished Fe bioavailability during oxygenation of the atmosphere (13), accompanied by Fe engagement in Fenton chemistry with O_2_^•−^. Furthermore, the appearance of modern cam-SODs that are able to utilize both Mn and Fe allows adaptation to a plethora of stresses, including oxidative stress and nutrient starvation, such as Mn scarcity (141, 148), representing elegant stress-resistance strategies (149) that are discussed later.

### The R2 subunit of class I RNRs

The RNRs are an ancient enzyme group uniquely responsible for the production of deoxyribonucleotides (dNTPs) from ribonucleotide precursors (150, 151). Different cation pair requirements have evolved in isoforms of the RNR R2 subunits. Fe or Mn cations are coordinated by this subunit, with the different metal ions facilitating activity in differing oxidative conditions. A high level of functional interchangeability between Fe or Mn cations has complicated the elucidation of the precise mechanism of the R2 subunit function. The chemically demanding redox reaction catalyzed by RNRs is essential in most organisms as a central controller of DNA replication, positioning these enzymes as potent targets for anticancer and antiviral drugs (152, 153). The implications of metal selection in these enzymes in pathogens are discussed later.

Three classes of RNR enzymes exist, all of which involve transition metal cofactors for radical generation and differ in their activity in aerobic or anaerobic environments. Class I RNRs, which are discussed here, are oxygen dependent and generate the catalytic radical *via* their dimetal binding R2 subunit.

The R2 subunits of class I RNRs are ferritin-like proteins (154, 155). These subunits contain a dinuclear metal binding site where the radical species are generated (17, 156). Metals are incorporated as divalent cations and oxidized to higher oxidation states as part of the activation mechanism. In the prototypic *E. coli* enzyme of the RNR family, the R2 subunit coordinates two Fe ions, defining class Ia subgroup, which also includes the human and yeast RNRs. The mechanism utilizes the electrons of the bound di-Fe in the reduction of molecular oxygen, resulting in an active state with a tyrosine radical (Tyr122) and an oxidized Fe^3+^ ion pair (17). While Mn^2+^ can bind in place of Fe^2+^, it does not support catalytic activity (157).

R2 proteins from other organisms have since been found to employ a pair of Mn ions (class Ib) or a “heterobimetallic” mixed Mn/Fe cofactor (class Ic) (158, 159, 160). While the di-Fe R2 proteins are damaged by H_2_O_2_, the Mn/Fe-binding R2 protein from the human pathogen *Chlamydia trachomatis* is resistant, and, in fact, becomes oxidized and activated in the presence of H_2_O_2_ (161). Interestingly, the *C. trachomatis* R2 subunit also has significant adaptations to the residues involved in the transfer of the radical from the metal site to the active site, lacking the highly conserved tyrosine, which usually harbors the radical following oxidation. It was proposed that because of the differing redox properties of the Mn in the metal ion pair, Mn can exist in an Mn^2+^ state upon oxidation and fulfill the oxidant function of the tyrosine radical. At the same time, Fe is unable to participate (162).

An interesting structural feature of the R2 proteins from the Mn/Fe or Mn/Mn binding RNRs allows selective self-assembly with the appropriate cations, despite the generally higher concentration of Fe in cells and the higher predicted stability of Fe^2+^ complexes over Mn^2+^ complexes (160, 163). Minor changes to coordinating residues can switch the binding preference between the two transition metals. A single mutation of a residue in the second coordination sphere alters the specificity of class Ib R2 subunit of *Bacillus anthracis*, such that under aerobic conditions, the protein is populated with an Mn/Fe ion pair (163).

The investigation of R2 proteins, which natively coordinate a di-Mn cofactor, proved problematic. Despite *in vivo* evidence that Mn was required, attempts to reconstitute the enzyme with Mn *in vitro* produced no activity, whereas introducing Fe was able to restore some enzymatic capacity. This is due to a requirement of the di-Mn mechanism for an additional cofactor, a flavin-mononucleotide coenzyme NrdI, which is present in all organisms expressing class Ib RNR. The oxidation of bound Mn^2+^ or Fe^2+^ is required for the activation of the R2 protein, and whereas Fe^2+^ reacts directly with O_2_ to become oxidized, Mn^2+^ requires NrdI to produce the oxidizing radical (164).

### Ribosomal Mg is a subject to replacement

As discussed earlier, the geological data indicate that the changes in earth's atmosphere and metal availability correlate with the biological utilization of metal ions by various biomolecules, including ribosomes (Fig. 3). Similarly to ancient SOD enzymes that appeared early during earth's evolution and underwent a significant divergence over time, the ribosomal core serves remarkably well as a tool to investigate early molecular biology and biochemistry, as it remained largely invariant since the last universal common ancestor (165), yet appeared to go through significant changes in respect of the utilization of metal ions (20, 166).

The contemporary ribosome coordinates Mg^2+^ extensively for structural stabilization, with X-ray analysis indicating ∼200 Mg^2+^ ions are associated with the large subunit alone (4), and further studies indicating as many as 1000 Mg^2+^ sites on the entire ribosome (167). At least six distinct Mg^2+^ binding structures were evident (20, 167), aiding in folding and assembly of the rRNA (168), mediating interactions with tRNA, mRNA, and stabilizing the intersubunit interface (169). Mg ions maintain a kink between the P-site and the A-site of the ribosome (170), and microclusters of Mg^2+^ pairs within the large subunit stabilize the peptidyl transfer center (171).

The high level of conservation of the ribosome since its evolution 3 to 4 billion years ago (172) leads to the idea that Mg^2+^ may not have been the original cation utilized in ribosomal structures, which first appeared prior to oxidation of the environment with less abundant Mg^2+^ and more prevalent ions of Mn^2+^ or Fe^2+^ (20, 173). Direct proof for this hypothesis came from a recent study by Bray *et al.* (20), wherein an ancient earth's atmosphere was replicated in an anoxic chamber with a 98% Ar and 2% H_2_, and lyophilized ribosomes were reassembled in the presence of Mn^2+^ or Fe^2+^ instead of Mg^2+^ ions. This elegant approach demonstrated that ribosomes retained their translational competency when their structure was rebuilt *in vitro* in the presence of alternative rRNA-stabilizing cations, suggesting metals' interchangeability within a prokaryotic ribosome (20). The conservation of the ribosome may mean that Mn^2+^ or Fe^2+^ represent ancient binding partners, which remain functional in extant organisms. However, the physiological relevance is dependent on metal availability within the cell. In addition, the presence of oxidant species in the present environment, which would not have been a concern in an anoxic archaean earth, produces the risk of damage to RNA structures closely associating with Fe^2+^ by Fenton-induced ROS.

**Figure 5:**
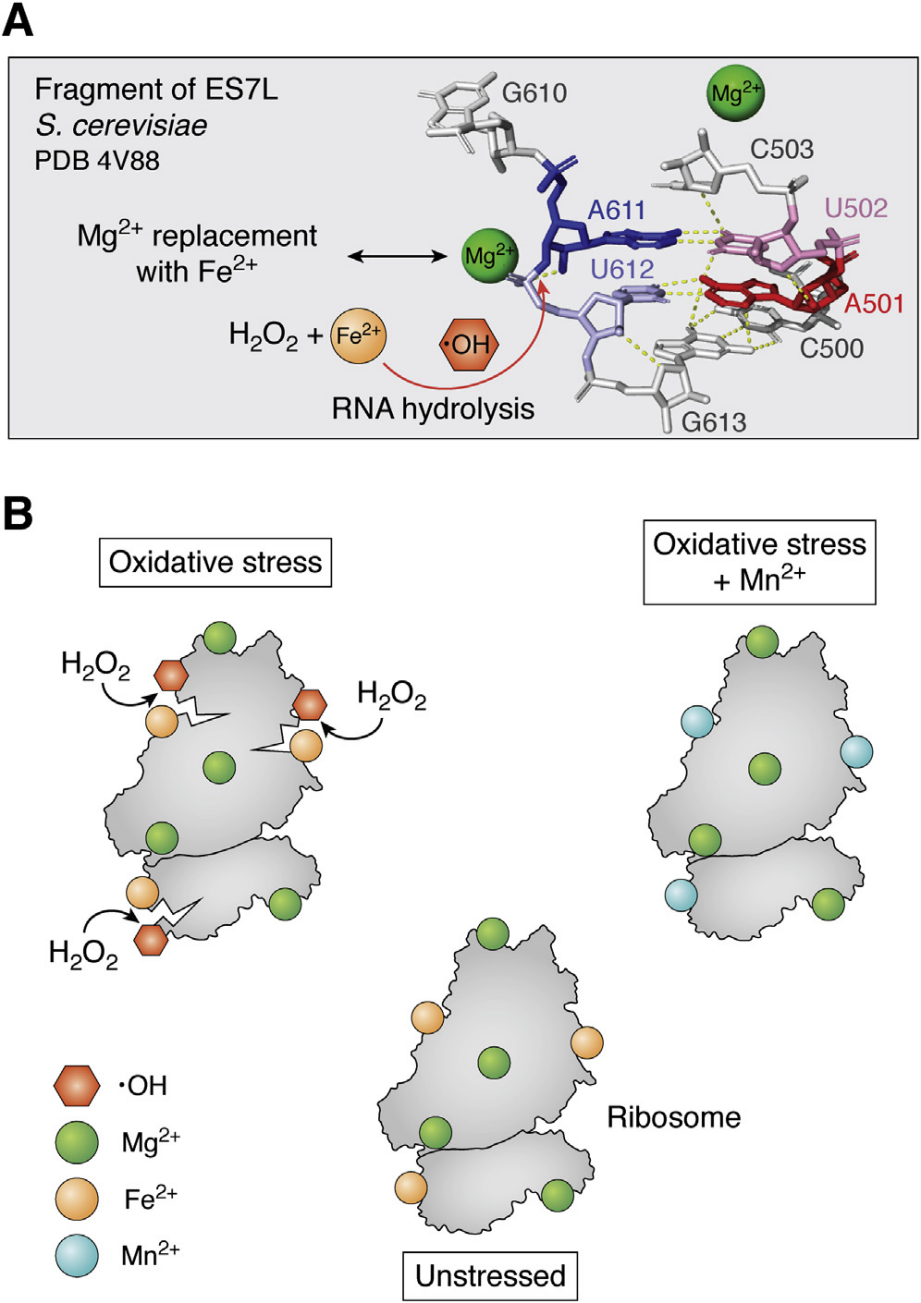
**rRNA is a substrate for the site-specific Fenton reaction.***A*, the highly conserved part of the expansion segment 7 (ES7L) of the large ribosomal subunit 60S from *Saccharomyces cerevisiae*. The depicted fragment corresponds to nucleotide pairs A501:U612 and U502:A611 of the ES7L and is shown in colors, surrounding bases are shown in *gray*. *Dashed lines* indicate predicted polar contacts between the four conserved bases (A501:U612 and U502:A611). Mg^2+^ ions are shown as *green spheres*. The Protein Data Bank file 4V88 was used. Replacement of Mg^2+^ with Fe^2+^ (shown as an o*range sphere*) powers the rRNA-localized Fenton reaction under oxidative conditions, whereupon the hydroxyl radical (•OH) is formed. •OH hydrolyses the sugar phosphate backbone at the specific site between A611 and U612. Modified from Ref. (21). *B*, Mg^2+^, Fe^2+^, and Mn^2+^ interchangeability on the ribosome. Unstressed cells contain active ribosomes, which bind divalent metal cations throughout their structure (*bottom*). These are predominantly Mg^2+^ but include a number of Fe^2+^ ions. In oxidative stress conditions, Fe^2+^ participates in Fenton reactions generating •OH radicals, which cleave the rRNA and fragment the ribosome (*top left*). However, if sufficient Mn^2+^ is available, the Fe^2+^ is displaced and Mn^2+^ occupies these sites. Under oxidative stress, a ribosome that coordinates Mn^2+^ in place of Fe^2+^ is not subjected to the generation of hydrolytic radical species and, thereby, is resistant to the stress-induced damages (*top right*). Modified from Ref. (123).

Indeed, a genetic screen of the *S. cerevisiae* deletion strains conducted in our laboratory has identified *grx5Δ* and *yfh1Δ* strains as highly susceptible to oxidant-induced rRNA scissions (21). Both these deletion strains contain a high level of labile Fe (21, 123), which prompted investigation of the possibility that rRNA hydrolysis is accomplished *via* the site-specific Fenton reaction. By devising an *in vitro* assay, the rRNA cleavage pattern observed in oxidant-treated *grx5Δ* cells was recapitulated *in vitro* with ribosomes purified from wildtype cells grown under normal nutrient-rich conditions, Fe(NH_4_)_2_(SO_4_)_2_ and ascorbic acid used as a prooxidant. The intensity and number of rRNA cleavage events were dependent on a concentration of Fe present in the *in vitro* reaction, as well as in strains carrying various levels of labile Fe, and correlated with cell viability. Interestingly, treatment of ribosomes with ascorbic acid alone still resulted in low-level cleavage within the expansion segment 7 of the large ribosomal subunit 60S (ES7L) of 25S rRNA (Fig. 5*A*), suggesting that even under normal growth conditions, ribosomes retained an ability to replace Mg^2+^ with Fe^2+^ at the selected sites. Similar low-intensity ES7L 25S rRNA cleavage has been detected upon treatment of cells with low doses of H_2_O_2_ (a condition, wherein H_2_O_2_ functions as a signaling molecule (174)), thus promoting resistance to subsequent acute oxidative stress (175). The Fe-dependent ES7L cleavage did not affect the translational activity of ribosomes, further suggesting a role of Mg^2+^-to-Fe^2+^ replacement within ES7L in the adaptive response to stress (175). Structural data (21) demonstrated that 2 Mg^2+^ ions exposed to the solvent side are located ∼6 to 8 Å away from the ES7L cleavage site (A611↓U612), implying that •OH radical generated during the Fenton reaction is in close proximity to the sugar phosphate backbone (Fig. 5*A*). Furthermore, we identified that Fe^2+^-mediated oxidant-dependent rRNA hydrolysis (175) was mitigated by increased Mn^2+^ availability, which seemingly can compete with Fe^2+^ for binding sites, resists induction of the Fenton reaction and, thus, accomplishes a protective role (123). The impact of Mn and Fe binding to ribosomes is outlined in Figure 5*B*. These results were consistent with the protective effect of replacing Fe^2+^ with Mn^2+^ described in *E. coli* under oxidative stress (176, 177).

Taken together, it seems reasonable to propose that the translation machinery maintained an ability to associate with the transition metal cations that stabilized its structure. Given that eukaryotic ribosomes retained the capability to replace Fenton-resistant Mg^2+^ with Fenton-active Fe^2+^ through the course of evolution, it is possible that this newly identified quality of the protein-translating machinery plays a regulatory role during gene expression as a means of adjustment to environmental changes.

The work described in this section was conducted and published recently, and, therefore, many questions have remained unanswered. It will be important to map other cleavage sites, dissect the molecular mechanics of stress adaptation accomplished by Fenton-cleaved ribosomes, investigate site-specific Fenton cleavages within human ribosomes, and elucidate human disease relevance. Furthermore, we would like to highlight that the discovery of metal interchangeability within the ancient molecular structure of the ribosome identifies an overlooked yet promising model molecule to study the evolution of biomolecules. Such observations recontextualize the ribosome, which is often considered as a stable entity resistant to changes or environment-induced perturbations, as being subject to metal ion replacement depending on genetically or environmentally induced changes in metal homeostasis. Thus, researchers should take special considerations while studying fundamental processes, such as translation and translational control.

The largely invariant core of the ribosome and the persistence of the translation machinery since the last universal common ancestor are central to many hypotheses regarding cellular evolution (172, 178). The oldest and most conserved part of the ribosome is free of protein and supports an early phase of biology, which was dominated by RNA. It has been demonstrated that RNA species are capable of carrying out Fe and Mn-mediated redox mechanisms (179, 180). Under anoxic conditions, a single-electron transfer reaction, much like those fundamental to metabolism, can be performed by RNA coordinating Fe^2+^ instead of Mg^2+^ (180). It is possible to substitute Mn^2+^ for Mg^2+^ in the hammerhead ribozyme, which is a small self-cleaving RNA that catalyzes reversible cleavage and ligation reactions at a specific site. Using 2′-mercaptonucleosides as biochemical probe and the “metal specificity switch” approach, it was found that Mn^2+^-to-Mg^2+^ substitution enhances enzymatic activity of the hammerhead ribozyme (179). These findings indicate the capability of RNA molecules to associate with a greater range of metal ions and possess more diverse catalytic abilities than are frequently observed in extant biology.

## Interchangeability with other metal ions

While much of the research on this topic focuses on Mn and Mg replacement by Fe at metal binding sites, flexible binding certainly extends to other biologically relevant metal ions. Zn is a widely utilized metal ion in proteins and may have replaced Fe as a cofactor in many enzymes where it presents less of a risk of toxicity (37).

The Zn finger (ZF) domain represents one of the most widespread and diverse structural motifs in biology, interacting with nucleic acid, protein, and lipid targets (181), and the bound Zn cation can be subject to replacement, such as in the estrogen receptor (182, 183). In this enzyme, Cu or Ni substitution results in loss of function, whereas DNA binding capacity is retained with Co or cadmium (183). In the case of Cu, this example illustrates the potential for damage caused by uncontrolled Cu availability as it can deactivate enzymes by displacement of Zn, in addition to its contribution to the generation of •OH radicals. In addition, replacement of Zn^2+^ with Fe^2+^ in the ZF domain of the estrogen receptor does not abolish DNA binding but can induce generation of oxidative radicals and cause DNA damage (184). The impact of metal ion replacement in ZF domains was recently reviewed in detail in Ref. (181).

Another interesting example of Zn^2+^-to-Fe^2+^ replacement was recently shown for the well-conserved scaffold protein ISCU (Iron–Sulfur Cluster assembly enzyme) that is central in 2Fe–2S and 4Fe–4S cluster synthesis and maturation, and, thus, has been the subject of extensive investigation (reviewed in Ref. (185)). Bound to Zn^2+^, Zn-ISCU catalyzes Fe–S clusters assembly inefficiently, whereas replacing Zn^2+^ with Fe^2+^, which can participate in an alternative redox-dependent reaction, generates “Fe-loaded” ISCU with a robust enzymatic activity (186). Gervason *et al.* (186) proposed that Fe-ISCU is the physiologically relevant form of the enzyme; however, further studies are required to dissect two mechanisms of Fe–S cluster assembly governed by Zn and Fe-loaded ISCUs. Nevertheless, this work revealed that, unlike for the ZF domain of the estrogen receptor (discussed previously (184)), replacement of Zn^2+^ with Fe^2+^ within ISCU might play a beneficial role in cellular physiology (186, 187).

In the Cu/Zn-SODs, the Cu ion plays the catalytic role, whereas Zn has a structural role, although it can be also required for catalytic activity that is maintained over a wide pH range (188, 189, 190). Cu ions have been shown to occupy empty Zn-binding sites within an SOD dimer, potentially inhibiting activity of the enzyme ((191) and Table 1). More recently, new members of this SOD class adapted to limited Zn availability conditions have been identified. These Cu-only SODs, found in both prokaryotes and eukaryotes, have an enhanced dimer interface, which provides stability and leaves them unable to bind Zn (192, 193), whereas other amino acid adaptations (Glu110) fulfill the electrostatic role of Zn (194).

Other metals with unpaired d-electrons, which have roles in biological systems, such as Co and Ni, may also replace Mg or Mn. Ni is not widely used as a cofactor in extant biochemistry, although it may have been an important catalyst in early biology. Within the small number of known Ni metalloenzymes are several interesting examples (reviewed in Ref. (195)), including Ni-SODs that are inactive upon Ni^2+^ replacements ((196, 197) and Table 1) and a conserved acireductone dioxygenase of the methionine salvage pathway, which binds either Ni^2+^ or Fe^2+^, and has distinct catalytic activities depending on the cofactor (198). Other examples are isoforms of mandelate racemase that prefer Mg but can also function with several other cation alternatives, including Mn, Ni, or Co (95, 199).

## Adapting to changes in metal availability

Pathogenic bacteria depend on their antioxidant systems as they aim to maintain a foothold in the chemically challenging host environment. The distinct families of SOD can be employed in combination to facilitate an effective antioxidant defense through changing metal availabilities, or as in some examples described earlier, a single isoform can be functional with multiple metal ions, conferring a similar adaptability. The *Streptomyces* genus contains Ni-SODs, sometimes along with an Fe-SOD isoform, which is expressed only when Ni is unavailable (121, 200). In many clinically relevant strains, the Fe-containing SOD has been lost and only the Ni-SOD remains, possibly reflecting the advantage of lowering requirements for Fe in mammalian pathogens to avoid host-imposed Fe restrictions (121, 201).

### Protective roles for Mn

The effect of metal limitation can be minimized by increased uptake. This is seen in *S. aureus* with two types of Mn uptake proteins, the ABC transporter MntABC and the Nramp-related MntH, which compete for Mn and enhance pathogen survival (202). Interestingly, these transporters are physiologically highly selective of Mn over Fe and other divalent cations (99), whereas Nramp family transporters are often associated with broad metal ion transport function. The exception to the Mn^2+^ specificity of MntH is related to the transport of Cd^2+^, which is also imported and contributes to toxicity (203), and which also inhibits the ABC-type Mn transporters (204), illustrating the challenges in selecting for strict metal targets. Studies in *E. coli* showed that MntH expression is regulated by OxyR as part of a battery of oxidative stress response genes that enhance Mn availability and limit Fe-mediated damage. These include catalase and peroxidase enzymes, the ferritin-like protein Dps that sequesters Fe^2+^, the Fe-uptake repressor Fur (205). It was shown that the activity of Mn-SOD is dependent on Mn import by MntH, and in unstressed cells, the low level of the transporter is a limiting factor of the activity of the SOD enzyme (205).

Mn-SOD from *E. coli* and human can also bind Fe^2+^ when it is present in excess relative to Mn^2+^. This metal cofactor replacement results, however, in formation of inactive Fe-bound enzyme ((206, 207) and Table 1). It was suggested that the cell's ability to appropriate Mn confers resistance through protection of numerous metalloproteins, which normally bind Fe^2+^ (177, 205). Another example for the selection of a particular metal ion by biosystems is an increased sensitivity of the pentose phosphate pathway to H_2_O_2_ that occurs because of the inactivation of an Fe-coordinating enzyme, ribulose-5-phosphate 3-epimerase (Rpe). It was shown that the enzyme-bound Fe^2+^ ion dissociates upon oxidation. Although restoring Fe^2+^ back to the binding site returns activity to most Rpe enzymes, a subset is damaged by the oxidation. This ultimately leads to total loss of Rpe activity through successive cycles of Fe binding and oxidation. The stress-induced increase in Mn import mitigates this mechanism, as Mn^2+^ can bind to and activate Rpe in place of Fe^2+^ without being sensitive to the presence of H_2_O_2_ (177). *E. coli* increase intracellular Mn^2+^ levels by over 10-fold in response to H_2_O_2_ (205), indicating that metal cofactor selection may be mediated by cellular control of relative concentrations. Interestingly, *in vitro* experiments showed that Zn^2+^ binds Rpe with greater affinity than Mn^2+^ or Fe^2+^ and is also present in the cell at higher concentrations. Thus, it is hypothesized that the wealth of other Zn^2+^-competing ligands in the cell, including glutathione, effectively reduces the availability of Zn to Rpe and other enzymes (177). Subsequent work established that this is not a unique case, as further nonredox metalloenzymes, which are likely to coordinate Fe^2+^ *in vivo*, are damaged by H_2_O_2_ and protected by increased Mn^2+^ import conducted by MntH, as well as by sequestration of Fe^2+^ by Dps (176).

The Mn-utilizing class Ib RNRs are well represented in pathogenic bacteria, including *B. anthracis*, *S. aureus*, and *E. coli* (158, 208), and likely arose through an evolutionary process of adaptation to oxidative stress and Fe restriction inflicted by hosts. Indeed, many human pathogen genomes encode RNRs from at least two classes (209, 210, 211). For example, the opportunistic pathogen *Streptococcus sanguinis* requires its class Ib RNR and Mn for aerobic growth and virulence. In contrast, class III RNR, which depends on an Fe–S cluster, is necessary for growth under anaerobic conditions (211).

### Metal-free alternatives

An alternative response to circumvent metal limitation involves utilizing surrogate proteins, which can substitute for the usual metal-dependent species and maintain activity while forgoing the metal entirely. The ribosome has been linked to the response to Zn limitation in bacteria (212). In response to Zn depletion, a number of Zn-binding ribosomal proteins are replaced with Zn-free homologs, making a significant contribution to the available Zn content of the cell (212, 213), although, perhaps, with a loss of translational activity (214). Other similar examples of metal-free homologs include an Mn-independent variant of phosphoglycerate mutase expressed in *S. aureus*, which allows glycolysis to continue during host-imposed Mn limitation (215).

Cu-only SODs are present in the pathogens *Mycobacterium tuberculosis* and *Candida albicans*, where they contribute to virulence by detoxification of host-derived oxidant species (216, 217). The Cu binding site is also more open than in the Cu/Zn-SODs, and in *C. albicans*, the enzyme is secreted prior to binding the metal ion, whereas eukaryotic extracellular Cu/Zn-SODs are usually charged with Cu and Zn within the cell. The combination of independence from Zn availability and the capability to bind extracellular Cu ions may be advantageous in maintaining catalytic activity (217).

### Bacterial infection and host metal sequestration mechanisms

Transition metals are essential for microorganisms like bacteria; thus, during nutrient limitation, mechanisms for metal acquisition become critical for bacterial cell survival. To overcome metal scarcity imposed by bacterial hosts, bacteria have evolved an elegant mechanism of synthetizing metal ion scavengers, known as metallophores, that possess high affinity to metal ions. Metallophores belong to a family of small molecules that bind various metal ions in the extracellular environment, following by active import of chelated metal complexes inside the bacterial cells. Normally, metallophores are divided into different groups based on their affinity toward a specific metal, such as siderophores for Fe, chalcophore for Cu, manganesophore for Mn, nickelophore for Ni, and zincophore for Zn ((218), references therein). Staphylopine produced by the pathogenic bacteria *S. aureus* stands out as a broad-spectrum affinity metallophore, as it is able of chelating various transition metals (219), thus efficiently overpowering host immunity.

Another nutrient limitation–induced strategy employed by the innate immune system involves calprotectin, which is released at infection sites from epithelial cells and neutrophils (212, 220). Calprotectin has previously been characterized as a chelator of Mn and Zn, while recent work establishes that it also actively sequesters Fe, and calprotectin-treated media were found to have reduced availability of all three metals (221). Neutrophils can contain very high calprotectin levels, sometimes accounting for as much as half of all protein content of the cytoplasm (222), which indicates the utility of the broad antimicrobial effect of metal sequestration. Proteins related to calprotectin of the S100 family of calcium-binding proteins are also important in host defense (27).

The metal sequestering action of calprotectin is exploited for competitive advantage in the gut pathogen *Salmonella typhimurium*, which expresses a high-affinity Zn transporter during infection. This allows it to survive the Zn restriction imposed by the host, whereas other commensal bacteria of the microbiota are more sensitive, resulting in reduced competition at the intestinal mucosa (223). The ability to adapt is particularly important for the transition between the different host environments exploited by opportunistic pathogens, such as group A *Streptococcus*. Recent work has highlighted the importance of metal homeostasis for survival of these bacteria in host organisms. The role of metals in the antioxidant defenses is important even while in a nonpathogenic state but becomes critical when the bacteria become invasive and induces an immune response (26, 30). The activity of calprotectin during *S. aureus* infection leads to a reduction in pathogen SOD activity, increased bacterial O_2_^•−^ levels, and improved clearance by the host immune system (148). The metal binding site of calprotectin is required for the antimicrobial effect, whereas supplying excess Mn^2+^ protects the pathogen against oxidative stress (148).

### Metals in viral infections

Viral genome replication and protein synthesis require trace metals, and their availability promotes the expansion of viral populations. Fe, in particular, has been studied for its association with viral infections, including those of HIV-1, human cytomegalovirus, hepatitis B and C viruses, and herpes simplex virus 1. Some viruses (arenaviruses and mouse mammary tumor virus) have been shown to target Fe-rich cells by utilizing Fe-import machinery for cell entry or manipulate host Fe homeostasis to their benefit. The availability of essential metals, including Fe, Mn, and Zn, can also influence the progression or resolution of many viral infections. This can occur as a result of both the immune system effects on metals and their direct impact on viral pathogenicity (224, 225, 226). Much is still unknown about the relevance of trace metal ions to virus–host interactions. Computational analysis has indicated that Zn-binding and Mn-binding domains are prevalent in viral proteomes and that many viral metal-binding proteins target host metal homeostasis regulators (227). The relevance of metal ions to the immune response to the SARS-CoV2—a cause of an unprecedented COVID-19 pandemic—has been discussed recently (228). As such, serum Fe levels have been linked to mortality risk in SARS-CoV2 patients (229, 230). Based on sequence analysis, it has been suggested that the cytoplasmic tail of the SARS-CoV2 spike protein may interact with the Fe exporter ferroportin (231). Whether Fe dysregulation in these patients is a result of inflammation or a contributing factor to pathogenesis remains currently unclear (230). However, most viruses require Fe as part of their replication cycle, and beneficial effects have been observed by treating other RNA virus infections with Fe chelators or inducing ferroportin expression to increase Fe efflux. The possibility of targeting SARS-CoV2 by limiting Fe availability is under investigation (232). At least two viral proteins encoded by the severe acute respiratory syndrome coronaviruses genomes (that are conserved in SARS-CoV2) bind Mn and function with other divalent metal cofactors. These include an endonuclease Nsp15, which promotes immune evasion and has shown activity with coordinated Mg^2+^ (233). Furthermore, an essential RNA-dependent RNA polymerase (RdRp) Nsp12, which shares binding site homology with an RdRp from poliovirus, was active with a broad range of divalent ions, such as Mg^2+^, Mn^2+^, and Fe^2+^ (234). Recent work has found that the catalytic subunit Nsp12 of the Nsp12–Nsp7–Nsp8 complex is able to accommodate two Fe–S clusters in the Zn-binding sites. Moreover, it was found that anoxically purified Fe–S–Nsp12 RdRp complex is even more prominent in the RNA template binding capacity and the polymerase activity than that assembled aerobically with two Zn ions (235). Thus, SARS-CoV2 RdRp is a *bona fide* subject of metal interchangeability relevant to the COVID-19 pandemic. Interestingly, nitroxide-enforced oxidation of Fe–S clusters caused their disassembly and blocked SARS-CoV2 replication in cell culture, raising a possibility that these clusters may serve as preferable cofactors for the SARS-CoV2 RdRp (235). Further investigation into roles for metal replacement strategies in viral infection may be beneficial for developing both specific and broadly applicable antiviral treatments.

## Metal imbalances as a causative factor of human disease states and metal chelation therapy

As they are key elements of numerous cellular biosystems (12), metal ion imbalances contribute to human disease states of inherited or acquired origin, such as chronic or aging enforced. For example, Fe deficiencies manifest in different types of anemias, whereby insufficient amounts of consumed/available Fe result in low levels of hemoglobin leading to insufficient oxygenation of body organs (reviewed in Ref. (236)). Metal imbalances are linked to pathophysiology of cardiovascular diseases, the number one killer worldwide (237, 238). For example, Fe deficiency is widespread in heart failure patients (239), whereas excessive Fe is linked to atherosclerosis and coronary heart disease (240, 241). Zn deficiency manifests in some malignancies, multiple sclerosis, and sepsis, wherein Zn scarcity was found to shift metal ions homeostasis leading to Fe and Cu overload (242, 243). In inherited blood disorders, such as β-thalassemia and sickle cell disease, mutations in hemoglobin subunit beta prohibit accommodation of Fe causing Fe overload (244). Mutation in the *FXN* gene encoding frataxin, the protein involved in Fe–S cluster assembly (245), cause Friedreich's ataxia (spinocerebellar degeneration) that is also associated with Fe overload (246). Another autosomal recessive disease, the Wilson disease (also known as hepatolenticular degeneration), affects primarily the liver and basal ganglia of the brain, and is caused by mutations in the *ATP7B* gene and generation of defective Cu transport protein leading to Cu build up (247, 248). A growing body of evidence has revealed that metal imbalances, such as Fe and Cu overload (249), Zn deficiency (250), manifest in numerous neurodegenerative disorders, such as Parkinson's disease, Alzheimer's disease, and Huntington's disease and are linked to enhanced oxidative stress that causes protein aggregation.

Having recognized that metal ions overload is a causative factor of disease etiology and progression, molecules that possess high affinity for metal ions are used in clinic to trap and neutralize excessive or toxic metals. Thus, the metal chelation therapy (MCT) has been administered as one of the effective ways to fight transition metals' overload, along with poisoning of heavy metals (251). MCT has been developed and used in clinical practice since the 1970s. By forming a complex with metal ion through ionic and coordination bonding (detailed in Ref. (218)), metal chelators alter metals' chemical properties, making metal ions unavailable for biological activities within metabolic pathways. As such, deferiprone, deferoxamine, and deferasirox are used for the removal of Fe during treatment of thalassemia, myelodysplasia, and sickle cell anemia, and penicillamine is used to deplete excessive Cu during Wilson's disease therapy (252). Animal studies have revealed a power of EDTA as an MCT agent in treatment of diabetic cataract, a condition that is associated with oxidative damage of lens cells (253). In addition, recently developed panel of small molecules, known as neuropeptides, which are produced and released by neurons, has shown to be effective during treatments of neurodegenerative diseases, providing neuroprotection. Neuropeptides are able to chelate excessive ions of Fe and Cu and, therefore, reduce formation of metal-mediated amyloid aggregates (254). Several excellent recent reviews (251, 255) discuss metal ion chelators in great details in a context of disease treatments, including newly developed drugs that are currently in clinical trials (255).

## Conclusions and future directions

The idea of a preferred cation associated with metal binding sites on biomolecules is strictly context dependent, as many of the examples discussed previously show. In fact, the chemical environment exudes a significant influence on metal ion chemistry. Technical challenges are commonly encountered when investigating physiologically relevant associations of biomolecules with metals (198, 202, 256). In a setting where enzyme activity can be measured *in vitro*, the strategy of chelating metal ions and then reintroducing them back into the biomolecule's structure, followed by testing for activity, is highly informative.

Here, we provide a new perspective relevant to the investigation of metal-bound biomolecules in the context of the metal interchangeability phenomenon. As such, we advise researchers to consider the particular cation that provides the highest activity to a biomolecule and those that may be functionally interchangeable and allow any activity, as they may be physiologically relevant under circumstances where ratios of available metals are disrupted or during stress. In more complex examples, such as *in vitro* experimentation to probe metal requirements, it is not possible, and both purification techniques and structural analyses are limited in their capacity to elucidate physiological metal ion interactions. In addition, several examples indicate that expressing alternative isoforms of biomolecules can circumvent metal limitation or oxidant damage, either by coordinating alternative metals that reduce the risk of damage or by going without the metal component altogether. The recent work on binding multiple metal cations to the ribosome expands on the established adaptability of protein enzymes in utilizing metals. A mounting body of evidence demonstrates that numerous metal-binding proteins maintain an amazing ability to adapt to metal flux and oxidative stress. The ubiquitous and conservative nature of these proteins and the ribosome sheds further light on the evolutionary pressures and geochemical shifts, thus, defining the relationship between biological systems, oxygen, and metals.

Further work is needed to understand the role of metal substitution on ribosomal activity and stability, as disrupted protein synthesis has broad impacts on human health. Changes in metal homeostasis are associated with many human diseases, including anemias and neurodegenerative disorders, in addition to the impacts on many pathogenic species and immune responses described in this review. Of current relevance, there is promising potential in exploring whether metal ion replacements can combat the propagation of viral infections, such as SARS-CoV2. Future work should also consider that flexibility of metal coordination in biomolecules may be underestimated and could have a broad reach across biological systems. Investigation into possible roles for metal ion interchangeability in fine-tuning cell behaviors and responses may reveal novel adaptive mechanisms.

## Conflict of interest

The authors declare that they have no conflicts of interest with the contents of this article.
